# Validation of the molecular international prognostic scoring system in patients with myelodysplastic syndromes defined by international consensus classification

**DOI:** 10.1038/s41408-023-00894-8

**Published:** 2023-08-09

**Authors:** Wan-Hsuan Lee, Ming-Tao Tsai, Xavier Cheng-Hong Tsai, Feng-Ming Tien, Min-Yen Lo, Mei-Hsuan Tseng, Yuan-Yeh Kuo, Ming-Chih Liu, Yi-Tsung Yang, Jui-Che Chen, Jih-Luh Tang, Hsun-I Sun, Yi-Kuang Chuang, Liang-In Lin, Wen-Chien Chou, Chien-Chin Lin, Hsin-An Hou, Hwei-Fang Tien

**Affiliations:** 1 Division of Hematology, Department of Internal Medicine, National Taiwan University Hospital, Taipei, Taiwan; 2 Department of Internal Medicine, National Taiwan University Hospital Hsin-Chu Branch, Hsinchu, Taiwan; 3 Graduate Institute of Clinical Medicine, College of Medicine, National Taiwan University, Taipei, Taiwan; 4 Department of Medical Education and Research, National Taiwan University Hospital Yunlin Branch, Yunlin, Taiwan; 5 Department of Internal Medicine, National Taiwan University Hospital Yunlin Branch, Yunlin, Taiwan; 6 Tai-Chen Cell Therapy Center, National Taiwan University, Taipei, Taiwan; 7 Department of Pathology, National Taiwan University Hospital, Taipei, Taiwan; 8 National Taiwan University Hospital Cancer Center Branch, Taipei, Taiwan; 9 Department of Clinical Laboratory Sciences and Medical Biotechnology, College of Medicine, National Taiwan University, Taipei, Taiwan; 10 Department of Laboratory Medicine, National Taiwan University Hospital, Taipei, Taiwan; 11 Department of Internal Medicine, Far-Eastern Memorial Hospital, New Taipei, Taiwan

**Keywords:** Myelodysplastic syndrome, Myelodysplastic syndrome

## Abstract

Myelodysplastic syndromes (MDS) have varied prognoses and require a risk-adapted treatment strategy for treatment optimization. Recently, a molecular prognostic model (Molecular International Prognostic Scoring System [IPSS-M]) that combines clinical parameters, cytogenetic abnormalities, and mutation topography was proposed. This study validated the IPSS-M in 649 patients with primary MDS (based on the 2022 International Consensus Classification [ICC]) and compared its prognostic power to those of the IPSS and revised IPSS (IPSS-R). Overall, 42.5% of the patients were reclassified and 29.3% were up-staged from the IPSS-R. After the reclassification, 16.9% of the patients may receive different treatment strategies. The IPSS-M had greater discriminative potential than the IPSS-R and IPSS. Patients with high, or very high-risk IPSS-M might benefit from allogeneic hematopoietic stem cell transplantation. IPSS-M, age, ferritin level, and the 2022 ICC categorization predicted outcomes independently. After analyzing demographic and genetic features, complementary genetic analyses, including *KMT2A*-PTD, were suggested for accurate IPSS-M categorization of patients with *ASXL1*, *TET2, STAG2, RUNX1, SF3B1, SRSF2*, *DNMT3A, U2AF1*, and *BCOR* mutations and those classified as MDS, not otherwise specified with single lineage dysplasia/multi-lineage dysplasia based on the 2022 ICC. This study confirmed that the IPSS-M can better risk-stratified MDS patients for optimized therapeutic decision-making.

## Introduction

Myelodysplastic syndromes (MDS) comprise a heterogeneous group of clonal myeloid neoplasms characterized by cytopenia due to ineffective hematopoiesis, dysplastic hematopoietic cells, and recurrent chromosomal abnormalities. Patients with MDS have varied clinical outcomes, running from an indolent course to aggressive disease with rapid progression to acute myeloid leukemia (AML) [1, 2]. A risk-adapted treatment strategy is mandatory to avoid unnecessary toxicities from treatment in low-risk patients and improve outcomes by using more aggressive or novel therapies in high-risk patients.

Molecular alterations play pivotal roles in the pathogenesis of MDS [3] and some recurrent mutations are important predictors of clinical outcomes [4–8]. Several prognostic models, including the International Prognostic Scoring System (IPSS) [9], revised IPSS (IPSS-R) [10], World Health Organization Classification-based Prognostic Scoring System [11], and MD Anderson Prognostic Scoring System [12] have been developed. However, none of these scoring systems incorporates genetic alterations. Recently, in studying 2,957 patients under the aegis of the International Working Group for Prognosis in MDS, Bernard et al. proposed a clinical-molecular prognostic model, IPSS-Molecular (IPSS-M) that combines clinical parameters, cytogenetic abnormalities, and somatic mutations of 31 genes [13]. Six risk category schema was established that resulted in the reclassification of 46% of the patients from their original IPSS-R classifications. This model was validated in an external cohort of 754 Japanese patients with MDS. In addition, Wu et al. demonstrated that the IPSS-M has a greater survival predictive accuracy than the IPSS-R for patients ≥60 years [14]. In 2022, the WHO classification (fifth edition) emphasized the integration of clinical, molecular, and pathologic parameters into MDS diagnosis (WHO-2022) [15]. Additionally, the 2022 International Consensus Classification (ICC) recategorized myeloid neoplasms based on the introduction of new entities and refined the criteria of the existing diagnostic categories [16].

In this study, we aimed to validate the IPSS-M in an Asian cohort of the 2022 ICC-defined MDS [16] and identify patients who may benefit therapeutically from the novel risk classification model. We compared the prognostic power of the IPSS-M with that of the IPSS and IPSS-R. The impacts of allogeneic hematopoietic stem cell transplantation (allo-HSCT) on the different IPSS-M risk categories were also evaluated.

## Materials and Methods

Based on the 2022 ICC, 649 patients with primary MDS whose bone marrow samples were adequately cryopreserved for deep-targeted sequencing were consecutively recruited. They were further risk-classified using the IPSS-M, IPSS-R, and IPSS. Patients with a history of chemotherapy/radiotherapy or hematologic malignancies were excluded for cohort homogeneity as the mutational landscapes of primary and secondary MDS differ [17, 18]. IPSS-M calculations were performed using the web calculator (https://mds-risk-model.com/) [13] provided by Bernard et al. This study was approved by the Research Ethics Committee of the National Taiwan University Hospital and written informed consent was obtained from all participants in accordance with the Declaration of Helsinki (approval number: 201709072RINC and 202109078RINB).

Cytogenetic analyses were performed and interpreted based on the International System for Human Cytogenetic Nomenclature [19, 20]. TruSight myeloid sequencing panel (Illumina, San Diego, CS, USA) and the HiSeq platform (Illumina, San Diego, CA, USA) were used to analyze the alterations of 54 myeloid-neoplasm relevant genes [21] (Supplementary Table 1), as previously described [22, 23]. Five residual genes (*ETNK1*, *GNB1*, *NF1*, *PPM1D*, and *PRPF8*), defined using the IPSS-M model, were not included. Library preparation and sequencing were performed according to the manufacturer’s instructions. The median reading depth was 10550x. We used the catalog of somatic mutations in cancer database version 86, single nucleotide polymorphism database version 151, ClinVar, polymorphism phenotyping version 2, and the sorting intolerant from tolerant algorithm to evaluate the consequence of every variant. The variant analysis algorithm for diagnostic samples has been described in detail previously [24]. The cut-off value of variant allele frequency was 5% for diagnostic samples. Owing to the limitation of next generation sequencing (NGS), analysis of *FLT3*-ITD was performed via polymerase chain reaction (PCR), followed by fluorescence capillary electrophoresis and that of *KMT2A*-PTD, by PCR followed by Sanger sequencing [25, 26].

### Statistical analysis

The Mann–Whitney U test was used for continuous variables and Fisher’s exact test or the χ^2^ test was applied for discrete variables. Kruskal‒Wallis test was used to determine statistical differences between medians of three or more groups. Leukemia-free survival (LFS) was defined as the interval between the date of diagnosis and that of the last follow-up, documented leukemic transformation, or death from any cause, whichever occurred first. Overall survival (OS) was the interval between the date of diagnosis and the last follow-up or death from any cause, whichever occurred first. Survival curves were plotted using the Kaplan‒Meier analysis, and statistical significance was calculated using the log-rank test. The Cox proportional hazards model was used for univariable and multivariable analyses. HSCT was evaluated as a time-dependent covariate [27]. The model’s predictive accuracy was assessed using Harrell’s concordance index [28]. All *P* values were two-sided and considered statistically significant at <0.05. All analyses were performed with the IBM SPSS Statistics v23 for Windows, R statistical language v4.2.2 for Windows and *jamovi*. 2.3.12.

## Results

### Demographic features

Patients’ characteristics are presented in Table 1. The median age of patients with MDS at diagnosis was 66.6 years, and the male sex was predominant (63.0%). Based on the 2022 ICC, 74.7% of the patients were assigned to the MDS group, including MDS with deletion-5q (MDS-del(5q), *n* = 4, 0.6%), MDS with mutated *SF3B1* (MDS-*SF3B1*, *n* = 52, 8.0%), MDS, not otherwise specified with single lineage dysplasia (MDS, NOS with SLD, *n* = 111, 17.1%), or multi-lineage dysplasia (MDS, NOS with MLD, *n* = 152, 23.4%), MDS with excess blasts (MDS-EB, *n* = 141, 21.7%), and MDS with mutated *TP53* (*n* = 25, 3.9%), and 25.3% of the patients were reassigned to the MDS/AML group. Based on the 2016 WHO classification, most patients had high risk MDS, such as EB (50.7%), and a relatively lower number of patients had isolated del(5q) (MDS-del(5q), 0.6 %), MDS with ring sideroblasts and SLD (MDS-RS-SLD, 6.9%), or MDS-RS with MLD (MDS-RS-MLD, 4.0%) (Table 1), which was similar to previous reports in Asian countries [22, 29, 30]. In addition, the frequency of each gene mutation is shown in Supplementary Fig. 1. *ASXL1* mutation (20.7%) was the most common mutation, followed by *TET2* (14.6%), *SF3B1* (13.7%), *RUNX1* (13.0%), *STAG2* (12.8%), and *DNMT3A* (10.1%) mutations. Based on IPSS-R cytogenetic categories, most (63.8%) patients had good-risk karyotypes, including normal karyotype in 58% of the patients, while 14.6% patients had complex karyotype.

**Table 1:** Clinical characteristics of patients with myelodysplastic syndromes.

Clinical characters	Total (*n* = 649)	%/range	Clinical characters	Total (*n* = 649)	%/range
Sex			MDS-related genes	94	14.5
Female	240	37.0	MDS-related cytogenetics	12	1.8
Male	409	63.0	mutated *TP53*	37	5.7
Age (years)^*^	66.6	18.4–94.5	NOS	21	3.2
Laboratory data^*^			2016 WHO		
WBC, X 10^9^/L	3.30	0.6–32.39	MDS-5q	4	0.6
ANC, X 10^9^/L	1.50	0–23.48	MDS-SLD	95	14.6
Hb, g/dL	8.1	2.6–17.1	MDS-MLD	143	22.0
Platelet, X 10^9^/L	77	1–931	MDS-RS-SLD	45	6.9
BM blast (%)	4.4	0–19.5	MDS-RS-MLD	26	4.0
PB blast (%)	0	0–9	MDS-EB1	139	21.4
2022 ICC			MDS-EB2	190	29.3
MDS	485	74.7	MDS-U	7	1.1
del(5q)	4	0.6	Treatment		
mutated *SF3B1*	52	8.0	HMA	156	24.0
NOS, with SLD	111	17.1	Intensive chemotherapy	20	3.1
NOS, with MLD	152	23.4	Clinical trial	27	4.2
EB	141	21.7	HSCT	103	15.9
mutated *TP53*	25	3.9	Supportive care only	286	44.6
MDS/AML	164	25.3	Other treatment^†^	145	22.6

^*^Median (range).

^†^Other treatment: include low-dose cytarabine, rabbit-derived anti-thymocyte globulin, cyclosporine, danazol, eltrombopag, erythropoietin-stimulating agents, thalidomide, steroid, venetoclax-based therapy and oral chemotherapy.

*ANC* absolute neutrophil count, *AML* acute myeloid leukemia, *BM* bone marrow, *EB* excess of blasts, *Hb* hemoglobin, *HMA* hypomethylating agent, *HSCT* hematopoietic stem cell transplantation, *ICC* International Consensus Classification, *MDS* myelodysplastic syndromes, *MDS-5q* MDS with isolated del(5q), *MDS-RS* MDS with ring sideroblasts, *MDS-EB* MDS with excess blasts, *MDS-SLD* MDS with single lineage dysplasia, *MDS-MLD* MDS with multilineage dysplasia, *MDS-RS-SLD* MDS with ring sideroblasts and single lineage dysplasia, *MDS-RS-MLD* MDS with ring sideroblasts and multilineage dysplasia, *MDS-U* MDS, unclassifiable, *NOS* not otherwise specified, *PB* peripheral blood, *WHO* World Health Organization, *WBC* white blood cell count.

When the patients were classified based on the IPSS-M (Table 2 and Supplementary Table 2), 14.2%, 18.6%, and 29.4% had moderate high, high, and very high risk, respectively, whereas only 18 (2.8%) patients had a very low risk. Distribution of the IPSS-M, IPSS-R, and IPSS in patients with MDS or MDS/AML based on 2022 ICC is summarized in Supplementary Table 3–5 and Supplementary Fig. 2. Patients diagnosed with MDS/AML had higher risk features than those diagnosed with MDS (Supplementary Table 3). Most patients with MDS or MDS/AML with mutated *TP53* had very high-risk IPSS-M/R or high-risk IPSS, as described previously [26] (Supplementary Tables 4 and 5). Patients at higher-risk IPSS-M received disease-modifying treatments (hypomethylating agent [HMA], intensive chemotherapy, clinical trials, or HSCT) more frequently than those with lower-risk IPSS-M (46.5% vs. 17.6%, *P* < 0.001; Supplementary Table 2). Within the median follow-up time of 61.5 months, 23.9% of the patients experienced leukemic transformation and 49.5% died at the end of follow-up.

**Table 2:** Leukemia-free survival and overall survival of patients with myelodysplastic syndromes (*n* = 649), categorized by the IPSS-M, IPSS-R, or IPSS.

	Number (%)	Median LFS (months)	95% CI (months)	C index	Median OS (months)	95% CI (months)	C index
IPSS-M				0.738			0.730
Very low	18 (2.8)	155.7	61.1–250.3		156.0	60.9–251.1	
Low	132 (20.3)	185.5	100.3–270.7		185.5	100.3–270.7	
Moderate low	95 (14.6)	85.2	22.6–147.8		85.2	59.1–111.3	
Moderate high	92 (14.2)	50.6	21.0–80.2		57.6	29.2–86.0	
High	121 (18.6)	25.1	17.2–33.0		31.1	23.6–38.6	
Very high	191 (29.4)	7.8	6.6–9.0		12.5	9.9–15.1	
IPSS-R				0.710			0.712
Very low	22 (3.4)	162.1	99.5–224.7		162.1	99.5–224.7	
Low	170 (26.2)	155.7	78.8–232.6		156.0	84.5–227.5	
Intermediate	173 (26.7)	53.8	29.4–78.2		57.6	35.4–79.8	
High	141 (21.7)	16.0	10.9–21.1		21.6	15.4–27.8	
Very high	143 (22.0)	7.5	6.1–8.9		8.7	6.5–10.9	
IPSS				0.681			0.679
Low	106 (16.3)	162.1	79.0–245.2		162.1	78.6–245.6	
Intermediate-1	302 (46.5)	68.0	36.4–99.6		73.3	43.9–102.7	
Intermediate-2	167 (57.7)	11.2	8.5–13.9		17.2	14.0–20.4	
High	74 (11.4)	7.8	5.6–10.0		10.1	7.6–12.6	

*CI* confidence interval, *IPSS* International Prognostic Scoring System, *IPSS-M* Molecular International Prognostic Scoring System, *IPSS-R* Revised International Prognostic Scoring System, *LFS* leukemia-free survival, *OS* overall survival.

### Risk reclassification of patients with MDS using the IPSS-M

**Fig. 1:**
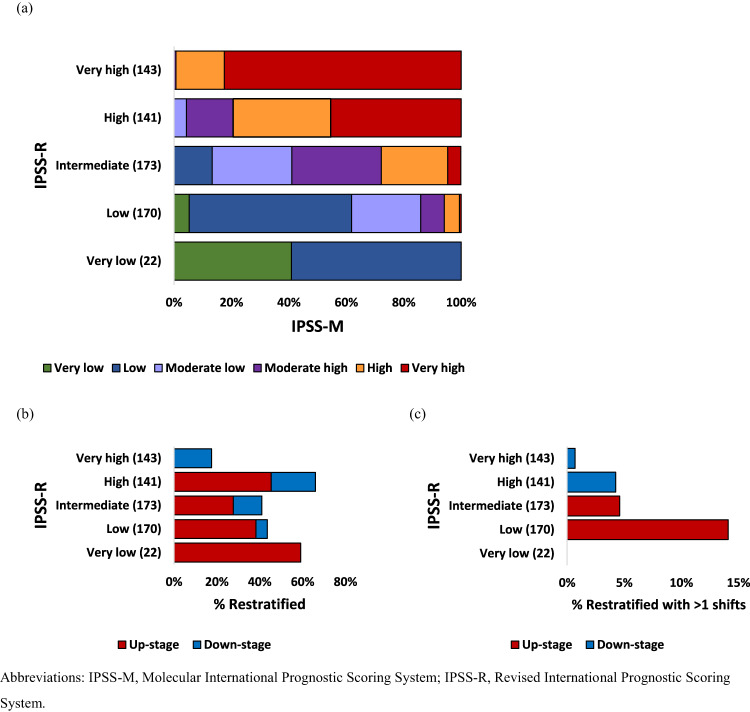
Comparison of the IPSS-M and IPSS-R. **a** Reclassification of the IPSS-R to IPSS-M for 649 patients with myelodysplastic syndrome. Each row corresponds to one IPSS-R category, and colors represent the IPSS-M categories. **b** Percentage of reclassified patients in each IPSS-R stratum, counting cases with any shift. **c** Percentage of reclassified patients in each IPSS-R stratum, counting cases with more than one shift.

As shown in Fig. 1a and Supplementary Table 6, by comparing the IPSS-M moderate low and moderate high-risk groups to the IPSS-R intermediate-risk group, 276 (42.5%) patients were reclassified. Of these, 190 (29.3%) cases were up-staged and 86 (13.2%) were down-staged (Supplementary Table 6). The percentage of the reclassified patients in each IPSS-R stratum is shown in Fig. 1b. Forty-five percent of the patients with high-risk IPSS-R were up-staged and 20.6% were down-staged. For those with intermediate-risk IPSS-R, 27.7% were up-staged to high (23.1%) or very high-risk (4.6%) IPSS-M, whereas 13.3% were shifted to the low-risk group. Moreover, 24% of patients were reclassified by more than one shift (Fig. 1c), with 24, eight, six, and one patient from the IPSS-R low, intermediate, high, and very high-risk groups, respectively. Among the reclassified patients, 48 (17.4%) had one mutated gene included in the IPSS-M, whereas 151 (53.7%) had two or more.

After the reclassification, 16.9% (110/649) of patients might receive different treatment strategies; of these, 15.3% (99/649) indicating for HMA or HSCT. We further evaluated the demographic features for these patients and found that 36.4% and 52.7% of them were classified as MDS, NOS with SLD (40/110), or MDS, NOS with MLD (58/110), respectively based on the 2022 ICC. Only one (0.9%) harbored *KMT2A*-PTD, whereas 15.5%, 12.7%, 10.9%, 9.1%, 9.1%, 7.3%, 7.3%, 7.3%, and 6.4% harbored mutated *ASXL1*, *TET2, STAG2, RUNX1, SF3B1, SRSF2*, *DNMT3A, U2AF1*, and *BCOR* respectively. (Supplementary Fig. 3).

### Prognostic values of the IPSS-M, IPSS-R, and IPSS in patients with 2022 ICC-defined MDS

**Fig. 2:**
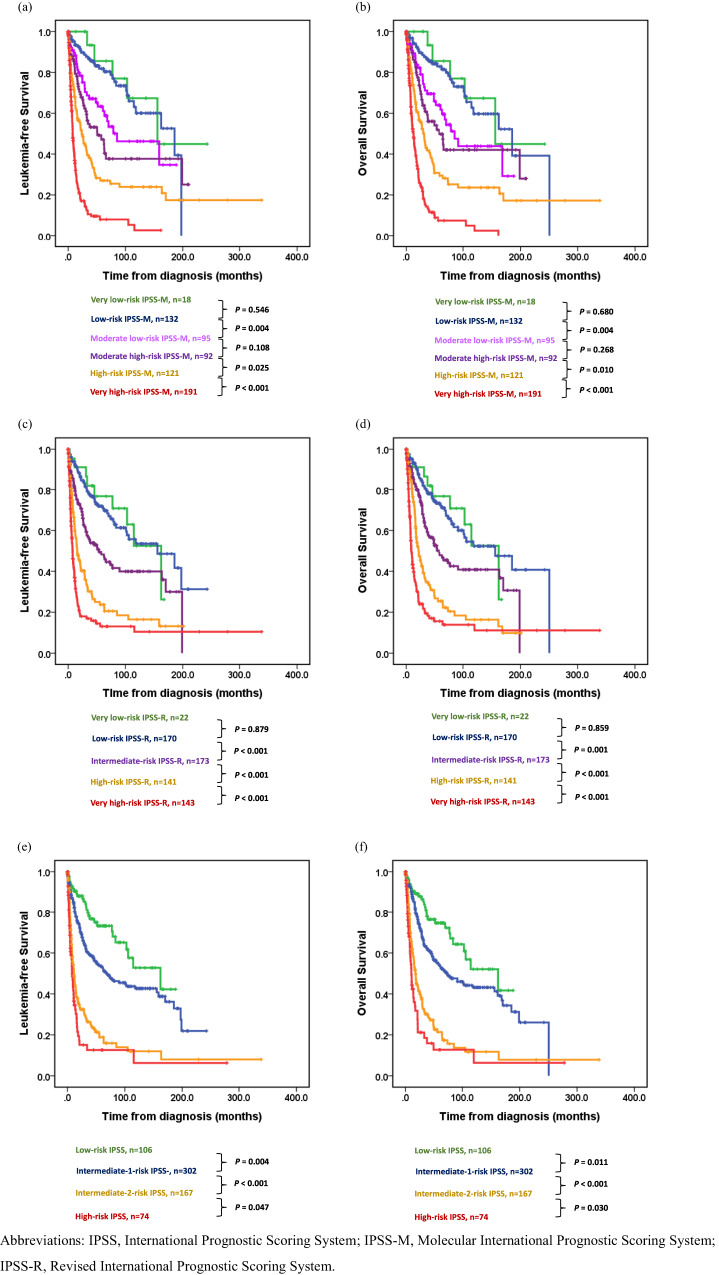
Kaplan‒Meier curves for leukemia-free survival and overall survival in patients with myelodysplastic syndrome, classified using the IPSS-M, IPSS-R, or IPSS. Leukemia-free survival (**a**) and overall survival (**b**), classified using the IPSS-M. Leukemia-free survival (**c**) and overall survival (**d**), classified using the IPSS-R. Leukemia-free survival (**e**) and overall survival (**f**), classified using the IPSS.

Cox regression analysis revealed that patients with MDS could be well stratified by the three systems for both LFS and OS. Hazard ratio (HR) were 1.91, 2.71, 4.01, and 10.93 in moderate low, moderate high, high, and very high-risk IPSS-M, respectively, for LFS and 1.91, 2.47, 3.91, and 10.05, respectively for OS compared with those of the very low/low-risk IPSS-M (Supplementary Table 7). Based on the IPSS-M model, the median LFS were 155.7, 185.5, 85.2, 50.6, 25.1, and 7.8 months in the very low, low, moderate low, moderate high, high, and very high-risk groups, respectively (Table 2; C-index 0.738), whereas the median OS were 156.0, 185.5, 85.2, 57.6, 31.1, and 12.5 months, respectively (Table 2; C-index, 0.730). Outcomes were comparable between patients with moderate low and moderate high-risk IPSS-M (Fig. 2). Subgroup analysis using the Kaplan‒Meier curves showed the discriminative power of the IPSS-M among different categories of the 2022 ICC (Supplementary Fig. 4). We evaluated the prognostic power of the three risk-scoring systems. The IPSS-M model could distinguish different risk categories more accurately than the IPSS-R and IPSS as indicated by the higher C-statistics (Table 2). Additionally, patients up-staged by the IPSS-M had inferior outcomes than those whose risk categories were unchanged within the same IPSS-R risk group (Supplementary Fig. 5). Furthermore, patients with very high-risk IPSS-R who were down-staged had longer survival than those whose risk categories were unchanged (Supplementary Figs. 5g, h). However, within each IPSS-M category, IPSS-R could not further stratify these patients. Only in the group of very high-risk IPSS-M, patients with high risk IPSS-R had better LFS and OS compared to those with very high risk IPSS-R (Supplementary Fig. 6).

According to a recent study [26] that explored the clinico-genetic features and prognostic implication of the 2022 ICC, the MDS-del(5q), MDS-*SF3B1*, and MDS, NOS with SLD/MLD were defined as low-risk MDS. In the univariable analysis, bedside IPSS-M, IPSS-R, IPSS, older age, male sex, and ferritin level were associated with poorer outcomes. In addition, the 2022 ICC had prognostic implications on LFS and OS. For the multivariable analysis, we adopted factors with *p* < 0.1 or factors that were clinically relevant in the univariable analysis (Supplementary Table 7) as covariates. Multivariable analysis showed that IPSS-M (*P* < 0.001), old age (HR for LFS: 1.028; for OS: 1.037, *P* < 0.001), high ferritin level (HR for LFS and OS: 1.013, *P* < 0.001) and the 2022 ICC categorization (*P* < 0.001) could predict LFS and OS independently and that HSCT could improve LFS (HR, 0.575, *P* = 0.039) (Table 3). Subgroup analysis of the impact of transplantation using time-dependent cox regression revealed that patients with high, or very high-risk IPSS-M benefited from allo-HSCT (HR for LFS: 0.43, and 0.52; HR for OS: 0.27, and 0.43, respectively, *P* < 0.05) (Supplementary Table 8 and Supplementary Fig. 7).

**Table 3:** Multivariable analysis Cox regression analysis of the impact of different variables on the leukemia-free survival and overall survival of patients with myelodysplastic syndromes.

Variable	LFS		OS	
	HR (95% CI)	*P* value	HR (95% CI)	*P* value
Age^*^	1.028 (1.018–10.38)	<0.001	1.037 (1.025–1.048)	<0.001
Female	0.757 (0.565–1.014)	0.062	0.774 (0.575–1.043)	0.093
Ferritin^*^(X 10^2^ ng/mL)	1.013 (1.008–1.019)	<0.001	1.013 (1.007–1.019)	<0.001
ICC^†^		<0.001		<0.001
Low-risk MDS^†^	Reference	–	Reference	–
MDS with EB	1.754 (1.130–2.722)	0.012	1.340 (0.856–2.097)	0.201
MDS/AML^‡^	2.011 (1.219–3.318)	0.006	1.601 (0.962–0.666)	0.070
Mutated *TP53*^§^	5.542 (2.978–10.312)	<0.001	6.206 (3.269–11.782)	<0.001
IPSS-M		<0.001		<0.001
Very low/low	Reference	–	Reference	–
Moderate low	1.576 (0.903–2.751)	0.110	1.709 (0.980–2.981)	0.059
Moderate high	2.324 (1.375–3.927)	0.002	2.211 (1.298–3.768)	0.004
High	2.871 (1.672–4.930)	<0.001	3.054 (1.783–5.232)	<0.001
Very high	5.259 (2.840–9.739)	<0.001	4.739 (2.544–8.827)	<0.001
HSCT	0.575 (0.341–0.971)	0.039	1.113 (0.702–1.763)	0.649

*P* values of <0.05 are statistically significant.

*As continuous variables analysis.

^†^Low-risk MDS included MDS with del(5q), MDS-*SF3B1*, and MDS, NOS with SLD or MLD.

^‡^MDS/AML with MDS-related gene mutations, MDS-related cytogenetic abnormalities, or not otherwise specified.

^§^MDS or MDS/AML with mutated *TP53.*

*CI* confidence interval, *EB* excess blasts, *HR* hazard ratios, *HSCT* allogeneic hematopoietic stem cell transplantation, *ICC* International Consensus Classification, *IPSS-M* Molecular International Prognostic Scoring System, *LFS* leukemia-free survival, *MDS* myelodysplastic syndromes, *MDS/AML* myelodysplastic syndromes/acute myeloid leukemia, *OS* overall survival.

The prognostic implication of the *SF3B1* mutation was equally validated. We confirmed that only *SF3B1*^α^ [13] conferred favorable prognosis and that patients with mutant *SF3B1*^β^ had similar outcomes with those with wild type *SF3B1* (*SF3B1*^β^ vs. wild type, LFS, 30.9 vs. 31.5 months, *P* = 0.702; OS, 36.3 vs. 36.4 months, *P* = 0.821). Patients with mutant *SF3B1*^5q^ (*n* = 2) had the shortest survival (LFS and OS, 1.6 months) (Supplementary Fig. 8).

## Discussion

Using a 2022 ICC-defined MDS cohort in Taiwan, we validated and compared the prognostic predictive power of the updated risk scoring system, IPSS-M with IPSS and IPSS-R and found that the IPSS-M is more advantageous than the other two systems. Using the IPSS-M model resulted in better discrimination of survival among each IPSS-R subgroup. Furthermore, we demonstrated that allo-HSCT could improve clinical outcomes in patients with high, or very high-risk IPSS-M.

The 2022 ICC provided important updates in the classification of hematological malignancies to facilitate accurate diagnosis, classification, and prognosis [16]. The main innovative changes in the ICC-defined MDS include the reclassification of MDS with blasts of 10–19% as MDS/AML, and the introduction of novel molecular-defining categories, including myeloid neoplasms with mutated *TP53*, and MDS/AML with MDS-related gene mutations. To the best of our knowledge, this is the first study to evaluate the prognostic implication of the IPSS-M based on the 2022 ICC-defined MDS, which revealed that aside from IPSS-M, the 2022 ICC categorization is equally an independent prognostic factor.

The IPSS-R has become a global standard for risk stratification, clinical trial enrollment, and treatment since its publication in 2012 [10]. It relies mainly on the severity of cytopenia, percentages of bone marrow blasts, and specific cytogenetic abnormalities. In the past decade, major advances to better understand the pathophysiology and molecular characteristics facilitated the development of risk scoring systems that integrate genetic alterations. Mounting evidence had demonstrated that the addition of mutation data improves the prognostic classification of patients with MDS [8, 22, 31–33]. Thus, under the coordination of several international institutions, the International Working Group for Prognosis in MDS provided a much larger combined database and a comprehensive prognostic system (IPSS-M) was developed with 2957 treatment-naïve MDS patients. The model encoded hemoglobin level, marrow blast percentage, and platelet count as continuous variables. Unlike the IPSS-R, the absolute neutrophil count was excluded from the model given its lack of independent prognostic value in the novel model. The IPSS-R cytogenetic categories were maintained [10, 34]. Molecular profiles included binary features of 16 main effect genes and a number of mutations from a residual group of 15 genes. Among them, multi-hit *TP53*, *FLT3*-ITD/TKD, and *KMT2A*-PTD played crucial roles in predicting adverse outcomes. This personalized prognostic model was more precise and resulted in the reclassification of nearly one-half (46%) of the patients from their original IPSS-R classifications. In this study, the rate of reclassification (42.5%) was numerically low, which might be resulted from the lower proportion of patients in the very low/low-risk IPSS-R groups compared to those of western countries (29.6% in this study vs. 56.9% [13], Supplementary Table 9). Among the reclassified patients, more than one-half (53.7%) of the patients had two or more mutated gene included in the IPSS-M. Therefore, the cumulative effects of the prognostic genes rather than those of the single gene were associated with the patient reclassification. Furthermore, strong and poor prognostic predictors, including *TP53* mutation, *FLT3* mutations, and *KMT2A*-PTD were identified in 10.0%, 0.8%, and 1.9% of our patients, respectively.

Notably, 6% of the IPSS-R very low/low groups in the discovery cohort recruited in the International Working Group for Prognosis were reclassified as IPSS-M very high/high-risk groups; however, none of our patients were so (Supplementary Table 6). Besides the study by Wu et al. [14], several conference papers [35, 36] confirmed the superior prognostic power of the IPSS-M over IPSS-R, which was similar to our results (Table 2). Nevertheless, application of the IPSS-M may be restrained from the different NGS platforms used by the individual institution that did not include all of the IPSS-M genes, especially *KMT2A*-PTD. Through the analyses of 2,876 patients with MDS from the GenoMed4All consortium, Elisabetta Sauta et al. [37] found that the information on the mutational status of a set of 15 genes (*ASXL1, CBL, DNMT3A, ETV6, EZH2, FLT3, IDH2, KMT2A-*PTD*, NPM1, NRAS, RUNX1, SF3B1, SRSF2, TP53*^multihit^, and *U2AF1*) could achieve 80% IPSS-M predictive accuracy. We validated this finding with 86.3% accuracy by using our cohort.

To date, there are limited data on the therapeutic implication of reclassification. Tariq Kewan et al. [35] suggested that although the IPSS-M may identify patients with different survival within individual IPSS-R subgroups, it may not contribute significant prognostic value in real-life scenarios involving patients receiving disease-modifying treatments. Similarly, Sandra Novoa Jáuregui et al. [36] found that only 9.5% of cases may benefit from IPSS-M reclassification owing to potential differences in clinical management, which was lower than expected. In this study, 99 (15.2%) patients were potential new candidates for disease-modifying treatments after the reclassification. Old age (>70 years) and/or multiple comorbidities hampered allo-HSCT in 36 patients, and the remaining 63 (9.7%) could potentially benefit clinically from IPSS-M reclassification. Thus, we explored the clinical and genetic characteristics of the IPSS-M reclassified patients. Patients with *ASXL1*, *TET2, STAG2, RUNX1, SF3B1, SRSF2*, *DNMT3A, U2AF1*, and *BCOR* mutations or patients classified as MDS, NOS with SLD/MLD based on the 2022 ICC, complementary genetic analyses including *KMT2A*-PTD were recommended for accurate IPSS-M classification, which would lead to different treatments strategies in a significant proportion (>5%) of the patients. A prospective large-cohort study is warranted to demonstrate the benefits of the new treatment policy in this subset.

The limitations of this study include that genetic aberrations in *ETNK1, GNB1, NF1, PPM1D* and *PRPF8*, which were considered as residual genes, were not analyzed. The mutational incidences of these five genes were low (<3%) as reported by Bernard et al. [13]. Accordingly, most of the patients in this study could be well categorized by the IPSS-M despite lack of mutation status of these five genes. Second, we could not validate the impact of IPSS-M in secondary or therapy-related MDS since the current study only enrolled de novo MDS. Nevertheless, the risks of patients with secondary or therapy-related MDS could be effectively assessed by utilizing the IPSS-M, allowing for appropriate stratification [13].

In conclusion, IPSS-M improved the prognostic accuracy and optimized treatments for patients with the 2022 ICC-defined MDS. Patients with high, or very high-risk IPSS-M might benefit from HSCT. In addition to the 2022 ICC, the IPSS-M provided an independent MDS prognosis. To facilitate the clinical implementation of IPSS-M, we identified the clinical and genetic characteristics of patients who might receive different therapies if classified with the new scoring system; however, further multicenter prospective studies are needed to confirm the application of the IPSS-M model.

## Supplementary information

Supplemental material

## Data Availability

The datasets generated during and/or analyzed during this study are available from the corresponding author upon reasonable request.
