# Supplementary material for: Validation of the molecular international prognostic scoring system in patients with myelodysplastic syndromes defined by international consensus classification
Source: Blood Cancer J. 2023 Aug 9;13(1):120. doi: 10.1038/s41408-023-00894-8 (PMC10412560; doi:10.1038/s41408-023-00894-8)
Supplement: Supplementary file 1 — Supplemental material [file 41408_2023_894_MOESM1_ESM.docx]

**Supplemental Table 1. List of 54 myeloid neoplasm-relevant genes studied in targeted NGS sequencing**

| **Gene name** | **Target region (exon)** | **Gene name** | **Target region (exon)** |
| --- | --- | --- | --- |
| ***ABL*** | 4-6 | ***JAK3*** | 13 |
| ***ASXL1*** | 12 | ***KDM6A*** | full |
| ***ATRX*** | 8-10, 17-31 | ***KIT*** | 2, 8-11, 13, 17 |
| ***BCOR*** | full | ***KRAS*** | 2, 3 |
| ***BCORL1*** | full | ***MLL*** | 5-8 |
| ***BRAF*** | 15 | ***MPL*** | 10 |
| ***CALR*** | 9 | ***MYD88*** | 3-5 |
| ***CBL*** | 8, 9 | ***NOTCH1*** | 26-28, 34 |
| ***CBLB*** | 9, 10 | ***NPM1*** | 12 |
| ***CBLC*** | 9, 10 | ***NRAS*** | 2, 3 |
| ***CDKN2A*** | full | ***PDGFRA*** | 12, 14, 18 |
| ***CEBPA*** | full | ***PHF6*** | Full |
| ***CSF3R*** | 14-17 | ***PTEN*** | 5, 7 |
| ***CUX1*** | full | ***PTPN11*** | 3, 13 |
| ***DNMT3A*** | full | ***RAD21*** | Full |
| ***ETV6*** | full | ***RUNX1*** | Full |
| ***EZH2*** | full | ***SETBP1*** | 4 (partial) |
| ***FBXW7*** | 9-11 | ***SF3B1*** | 13-16 |
| ***FLT3*** | 14, 15, 20 | ***SMC1A*** | 2, 11, 16, 17 |
| ***GATA1*** | 2 | ***SMC3*** | 10, 13, 19, 23, 25, 28 |
| ***GATA2*** | 2-6 | ***SRSF2*** | 1 |
| ***GNAS*** | 8, 9 | ***STAG2*** | full |
| ***HRAS*** | 2, 3 | ***TET2*** | 3-11 |
| ***IDH1*** | 4 | ***TP53*** | 2-11 |
| ***IDH2*** | 4 | ***U2AF1*** | 2, 6 |
| ***IKZF1*** | full | ***WT1*** | 7, 9 |
| ***JAK2*** | 12, 14 | ***ZRSR2*** | full |

**Supplemental Table 2. Clinical characteristics of patients (n=649) with myelodysplastic syndromes, categorized by the IPSS-M**

| Clinical characters | Very low  (n=18) | Low  (n=132) | Moderate low  (n=95) | Moderate  high  (n=92) | High  (n=121) | Very high  (n=191) | *P* value |
| --- | --- | --- | --- | --- | --- | --- | --- |
| Sex |  |  |  |  |  |  | 0.277 |
| Female | 7 (38.9%) | 47 (35.6%) | 44 (46.3%) | 38 (41.3%) | 39 (32.2%) | 65 (34.0%) |  |
| Male | 11 (61.1%) | 85 (64.4%) | 51 (53.7%) | 54 (58.7%) | 82 (67.8%) | 126 (66.0%) |  |
| Age^*^ | 61.5  (33.1-83.3) | 69.1  (20.6-94.5) | 67.1  (18.5-87.9) | 59.2  (19.3-94.2) | 63.9  (18.4-93.1) | 68.4  (23.6-94.1) | **0.018** |
| Laboratory data^*^ |  |  |  |  |  |  |  |
| WBC, ×10^9^ /L | 3.25  (1.25-15.82) | 4.17  (0.73-32.39) | 3.16  (0.66-26.31) | 2.80  (0.75-15.55) | 2.79  (0.72-32.39) | 3.31  (0.60-23.60) | **0.001** |
| ANC, ×10^9^ /L | 1.72  (0.44-8.39) | 2.20  (0.05-10.76) | 1.75  (0-15.65) | 1.30  (0.96-12.02) | 1.29  (0.01-23.48) | 1.11  (0.16-17.01) | **<0.001** |
| Hb, g/dL | 12.8  (10.7-17.1) | 9.1  (5.7-14.4) | 8.1  (4.5-13.6) | 7.7  (4.2-14.6) | 7.9  (3.2-13.5) | 7.6  (2.6-12.7) | **<0.001** |
| Platelet, ×10^9^ /L | 134  (20-370) | 156  (4-499) | 77  (7-471) | 88  (3-721) | 63  (1-655) | 63  (1-931) | **<0.001** |
| BM blast (%) | 2.0  (0.4-5.0) | 1.8  (0-8.2) | 1.8  (0-12.0) | 3.0  (0-17.3) | 6.2  (0-18.0) | 11.8  (0.5-19.5) | **<0.001** |
| PB blast (%) | 0 (0-1) | 0 (0-9) | 0 (0-4) | 0 (0-8) | 0 (0-9) | 1 (0-9) | **<0.001** |
| Treatment |  |  |  |  |  |  |  |
| HMA | 0 (0.0%) | 10 (7.6%) | 11 (11.6%) | 19 (20.7%) | 36 (29.8%) | 80 (41.9%) | **<0.001** |
| Intensive chemotherapy | 0 (0.0%) | 1 (0.8%) | 2 (2.1%) | 3 (3.3%) | 2 (1.7%) | 12 (6.3%) | **0.057** |
| Clinical trial | 2 (7.1%) | 4 (3.1%) | 4 (4.3%) | 2 (2.2%) | 5 (4.2%) | 10 (5.3%) | 0.501 |
| HSCT | 3 (16.7%) | 14 (10.6%) | 8 (8.4%) | 16 (17.4%) | 27 (22.3%) | 35 (18.3%) | **0.034** |
| Supportive care | 11 (61.1%) | 75 (58.6%) | 57 (60.6%) | 40 (43.5%) | 43 (36.1%) | 60 (31.6%) | **<0.001** |
| Other treatment^†^ | 6 (33.3%) | 37 (28.9%) | 21 (22.3%) | 29 (31.5%) | 25 (21.0%) | 27 (14.2%) | **0.006** |

*P* values of <0.05 are statistically significant.

*Median (range).

^†^Other treatment: include low-dose cytarabine, rabbit-derived anti-thymocyte globulin, cyclosporine, danazol, eltrombopag, erythropoietin-stimulating agents, thalidomide, steroid, venetoclax-based therapy and oral chemotherapy.

Abbreviations: ANC, absolute neutrophil count; BM, bone marrow; Hb, hemoglobin; HMA, hypomethylating agent; HSCT, hematopoietic stem cell transplantation; PB, peripheral blood; WBC, while blood cell count.

**Supplemental Table 3. Distribution of the IPSS-M, IPSS-R and IPSS in patients with myelodysplastic syndromes, or myelodysplastic syndromes/acute myeloid leukemia, categorized by 2022 International Consensus Classification**

| Variables | MDS  (n=485/74.7%) | MDS/AML  (n=164/25.3%) | *P* value |
| --- | --- | --- | --- |
| IPSS-M |  |  | **<0.001** |
| Very low | 18 (3.7%) | 0 (0.0%) | **0.010** |
| Low | 132 (27.2%) | 0 (0.0%) | **<0.001** |
| Moderate low | 91 (18.8%) | 4 (2.4%) | **<0.001** |
| Moderate high | 84 (17.3%) | 8 (4.9%) | **<0.001** |
| High | 92 (19.0%) | 29 (17.7%) | 0.715 |
| Very high | 68 (14.0%) | 123 (75.0%) | **<0.001** |
| IPSS-R |  |  | **<0.001** |
| Very low | 22 (4.5%) | 0 (0.0%) | **0.002** |
| Low | 170 (35.1%) | 0 (0.0%) | **<0.001** |
| Intermediate | 162 (33.4%) | 11 (6.7%) | **<0.001** |
| High | 78 (16.1%) | 63 (38.4%) | **<0.001** |
| Very high | 53 (10.9%) | 90 (54.9%) | **<0.001** |
| IPSS |  |  | **<0.001** |
| Low | 106 (21.9%) | 0 (0.0%) | **<0.001** |
| Intermediate-1 | 297 (61.2%) | 5 (3.0%) | **<0.001** |
| Intermediate-2 | 82 (16.9%) | 85 (51.8%) | **<0.001** |
| High | 0 (0.0%) | 74 (45.1%) | **<0.001** |

Note: Data presented in number (%).

*P* values of <0.05 are statistically significant.

Abbreviations: IPSS, International Prognostic Scoring System; IPSS-M, Molecular International Prognostic Scoring System; IPSS-R, Revised International Prognostic Scoring System; MDS, myelodysplastic syndromes; MDS/AML, myelodysplastic syndromes/acute myeloid leukemia.

**Supplemental Table 4. Distribution of the IPSS-M, IPSS-R and IPSS in patients with different subtypes of myelodysplastic syndromes, based on 2022 International Consensus Classification**

| Variables | del(5q)  (n=4) | mutated *SF3B1*  (n=52) | NOS,  SLD  (n=111) | NOS,  MLD  (n=152) | EB  (n=141) | mutated *TP53*  (n=25) | *P* value |
| --- | --- | --- | --- | --- | --- | --- | --- |
| IPSS-M |  |  |  |  |  |  | **<0.001** |
| Very low | 0 (0.0%) | 2 (3.8%) | 5 (4.5%) | 9 (5.9%) | 2 (1.4%) | 0 (0.0%) |  |
| Low | 0 (0.0%) | 34 (65.4%) | 44 (39.6%) | 46 (20.3%) | 8 (5.7%) | 0 (0.0%) |  |
| Moderate low | 0 (0.0%) | 12 (23.1%) | 29 (26.1%) | 33 (21.7%) | 17 (12.1%) | 0 (0.0%) |  |
| Moderate high | 2 (50.0%) | 4 (7.7%) | 20 (18.0%) | 31 (20.4%) | 27 (19.1%) | 0 (0.0%) |  |
| High | 1 (25.0%) | 0 (0.0%) | 11 (9.9%) | 23 (15.1%) | 57 (40.4%) | 0 (0.0%) |  |
| Very high | 1 (25.0%) | 0 (0.0%) | 2 (1.8%) | 10 (6.6%) | 30 (21.3%) | 25 (100%) |  |
| IPSS-R |  |  |  |  |  |  | **<0.001** |
| Very low | 0 (0.0%) | 7 (13.5%) | 9 (8.1%) | 6 (3.9%) | 0 (0.0%) | 0 (0.0%) |  |
| Low | 1 (25.0%) | 35 (67.3%) | 55 (49.5%) | 66 (43.4%) | 13 (9.2%) | 0 (0.0%) |  |
| Intermediate | 3 (75.0%) | 10 (19.2%) | 39 (35.1%) | 57 (37.5%) | 53 (37.6%) | 0 (0.0%) |  |
| High | 0 (0.0%) | 0 (0.0%) | 6 (5.4%) | 18 (11.8%) | 52 (36.9%) | 2 (8.0%) |  |
| Very high | 0 (0.0%) | 0 (0.0%) | 2 (1.8%) | 5 (3.3%) | 23 (16.3%) | 23 (92.0%) |  |
| IPSS |  |  |  |  |  |  | **<0.001** |
| Low | 0 (0.0%) | 27 (51.9%) | 48 (43.2%) | 24 (15.8%) | 7 (5.0%) | 0 (0.0%) |  |
| Intermediate-1 | 4 (100%) | 25 (48.1%) | 59 (53.2%) | 116 (76.3%) | 93 (66.0%) | 0 (0.0%) |  |
| Intermediate-2 | 0 (0.0%) | 0 (0.0%) | 4 (3.6%) | 12 (7.9%) | 41 (29.1%) | 25 (100%) |  |
| High | 0 (0.0%) | 0 (0.0%) | 0 (0.0%) | 0 (0.0%) | 0 (0.0%) | 0 (0.0%) |  |

Note: Data presented in number (%).

*P* values of <0.05 are statistically significant.

Abbreviations: EB, excess blasts; IPSS, International Prognostic Scoring System; IPSS-M, Molecular International Prognostic Scoring System; IPSS-R, Revised International Prognostic Scoring System; MLD, multilineage dysplasia; NOS, not otherwise specified; SLD, single lineage dysplasia.

**Supplemental Table 5. Distribution of the IPSS-M, IPSS-R and IPSS in patients with different subtypes of myelodysplastic syndromes/acute myeloid leukemia, based on 2022 International Consensus Classification**

| Variables | mutated *TP53*  (n=37) | MDS-related gene mutations  (n=94) | MDS-related cytogenetics  (n=12) | NOS  (n=21) | *P* value |
| --- | --- | --- | --- | --- | --- |
| IPSS-M |  |  |  |  | **<0.001** |
| Very low | 0 (0.0%) | 0 (0.0%) | 0 (0.0%) | 0 (0.0%) |  |
| Low | 0 (0.0%) | 0 (0.0%) | 0 (0.0%) | 0 (0.0%) |  |
| Moderate low | 0 (0.0%) | 3 (3.2%) | 0 (0.0%) | 1 (4.8%) |  |
| Moderate high | 0 (0.0%) | 2 (2.1%) | 1 (8.3%) | 5 (23.8%) |  |
| High | 0 (0.0%) | 18 (19.1%) | 3 (25.0%) | 8 (38.1%) |  |
| Very high | 37 (100%) | 71 (75.5%) | 8 (66.7%) | 7 (33.3%) |  |
| IPSS-R |  |  |  |  | **<0.001** |
| Very low | 0 (0.0%) | 0 (0.0%) | 0 (0.0%) | 0 (0.0%) |  |
| Low | 0 (0.0%) | 0 (0.0%) | 0 (0.0%) | 0 (0.0%) |  |
| Intermediate | 0 (0.0%) | 7 (7.4%) | 0 (0.0%) | 4 (19.0%) |  |
| High | 0 (0.0%) | 52 (55.3%) | 3 (25.0%) | 8 (38.1%) |  |
| Very high | 37 (100%) | 35 (37.2%) | 9 (75.0%) | 9 (42.9%) |  |
| IPSS |  |  |  |  | **<0.001** |
| Low | 0 (0.0%) | 0 (0.0%) | 0 (0.0%) | 0 (0.0%) |  |
| Intermediate-1 | 0 (0.0%) | 4 (4.3%) | 0 (0.0%) | 1 (4.8%) |  |
| Intermediate-2 | 2 (5.4%) | 65 (69.1%) | 1 (8.3%) | 17 (81.0%) |  |
| High | 35 (94.6%) | 25 (26.6%) | 11 (91.7%) | 3 (14.3%) |  |

Note: Data presented in number (%).

*P* values of <0.05 are statistically significant.

Abbreviations: IPSS, International Prognostic Scoring System; IPSS-M, Molecular International Prognostic Scoring System; IPSS-R, Revised International Prognostic Scoring System; NOS, not otherwise specified.

**Supplemental Table 6. Distribution of the IPSS-M in comparison to the IPSS-R.**

|  | IPSS-M | | | | | |
| --- | --- | --- | --- | --- | --- | --- |
|  | Very low  (n=18) | Low  (n=132) | Moderate low  (n=95) | Moderate high  (n=92) | High  (n=121) | Very high  (n=191) |
| IPSS-R (n=649) |  |  |  |  |  |  |
| Very low (n=22) | 9 (40.9%) | 13 (59.1%) | 0 (0.0%) | 0 (0.0%) | 0 (0.0%) | 0 (0.0%) |
| Low (n=170) | 9 (5.3%) | 96 (56.5%) | 41 (24.1%) | 14 (8.2%) | 9 (5.3%) | 1 (0.6%) |
| Intermediate (n=173) | 0 (0.0%) | 23 (13.3%) | 48 (27.8%) | 54 (31.2%) | 40 (23.1%) | 8 (4.6%) |
| High (n=141) | 0 (0.0%) | 0 (0.0%) | 6 (4.3%) | 23 (16.3%) | 48 (34.0%) | 64 (45.4%) |
| Very high (n==143) | 0 (0.0%) | 0 (0.0%) | 0 (0.0%) | 1 (0.7%) | 24 (16.8%) | 118 (82.5%) |

Note: Data presented in number (%).

Abbreviations: IPSS-M, Molecular International Prognostic Scoring System; IPSS-R, Revised International Prognostic Scoring System.

**Supplemental Table 7. Univariable Cox regression analyses for leukemia-free survival and overall survival**

| Variables | LFS | | OS | |
| --- | --- | --- | --- | --- |
|  | **HR (95% CI)** | ***P* value** | **HR (95% CI)** | ***P* value** |
| Age^*^ | 1.024 (1.017-1.032) | **<0.001** | 1.027 (1.019-1.034) | **<0.001** |
| Female | 0.684 (0.543-0.862) | **0.001** | 0.663 (0.523-0.840) | **0.001** |
| ICC |  |  |  |  |
| Low-risk MDS^†^ | Reference | - | Reference | - |
| MDS with EB | 2.773 (2.093-3.674) | **<0.001** | 2.490 (1.870-3.316) | **<0.001** |
| MDS/AML^‡^ | 3.974 (2.970-5.317) | **<0.001** | 3.562 (2.648-4.792) | **<0.001** |
| Mutated *TP53*^§^ | 14.674 (10.117-21.285) | **<0.001** | 17.823 (12.148-26.149) | **<0.001** |
| Ferritin^*^(x 10^2^ ng/mL) | 1.011 (1.007-1.016) | **<0.001** | 1.012 (1.007-1.017) | **<0.001** |
| LDH^*^ (U/L) | 1.030 (0.971-1.093) | 0.329 | 1.026 (0.966-1.090) | 0.403 |
| IPSS-M |  |  |  |  |
| Very low/low | Reference | - | Reference | - |
| Moderate low | 1.905 (1.191-3.044) | **0.007** | 1.906 (1.191-3.049) | **0.007** |
| Moderate high | 2.711 (1.751-4.196) | **<0.001** | 2.467 (1.579-3.856) | **<0.001** |
| High | 4.010 (2.695-5.965) | **<0.001** | 3.911 (2.627-5.824) | **<0.001** |
| Very high | 10.933 (7.503-15.931) | **<0.001** | 10.048 (6.886-14.661) | **<0.001** |
| IPSS-R |  |  |  |  |
| Very low/low | Reference | - | Reference | - |
| Intermediate | 1.854 (1.326-2.592) | **<0.001** | 1.725 (1.228-2.421) | **0.002** |
| High | 3.565 (2.569-4.947) | **<0.001** | 3.401 (2.446-4.728) | **<0.001** |
| Very high | 6.280 (4.548-8.671) | **<0.001** | 6.096 (4.403-8.439) | **<0.001** |
| IPSS |  |  |  |  |
| Low | Reference | - | Reference | - |
| Intermediate-1 | 1.801 (1.199-2.705) | **0.004** | 1.701 (1.131-2.558) | **0.010** |
| Intermediate-2 | 5.089 (3.359-7.712) | **<0.001** | 4.702 (3.098-7.136) | **<0.001** |
| High | 7.201 (4.505-11.508) | **<0.001** | 6.813 (4.249-10.925) | **<0.001** |
| HSCT | 0.776 (0.543-1.110) | 0.165 | 1.048 (0.771-1.425) | 0.765 |

Note: Only 18 patients (2.7%) were categorized as IPSS-M very low risk and there was no inter-group difference between IPSS-M very low and low risk subgroups in both OS and LFS; accordingly, we put IPSS-M very low and low groups together.

*P* values of <0.05 are statistically significant.

*As continuous variables analysis.

^†^Low-risk MDS includes MDS with del(5q), MDS-*SF3B1*, and MDS, NOS with SLD or MLD.

^‡^MDS/AML includes MDS/AML with MDS-related gene mutations, MDS/AML with MDS-related cytogenetic abnormalities, or MDS/AML, not otherwise specified

^§^MDS or MDS/AML with mutated *TP53*

Abbreviations: CI, confidence interval; EB, excess blasts; HR, hazard ratio; HSCT, hematopoietic stem cell transplantation; ICC, International Consensus Classification; IPSS, International Prognostic Scoring System; IPSS-M, Molecular International Prognostic Scoring System; IPSS-R, Revised International Prognostic Scoring System; LFS, leukemia-free survival; MDS, myelodysplastic syndromes; MDS/AML, myelodysplastic syndromes/acute myeloid leukemia; OS, overall survival.

**Supplemental Table 8. Time-dependent Cox regression subgroup analysis for the impact of allogeneic hematopoietic stem cell transplantation on leukemia-free survival and overall survival.**

| IPSS-M | LFS | | OS | |
| --- | --- | --- | --- | --- |
|  | **HR (95% CI)** | ***P* value** | **HR (95% CI)** | ***P* value** |
| Very low/low | 0.449 (0.106-1.898) | 0.276 | 0.950 (0.330-2.737) | 0.925 |
| Moderate low | 0.540 (0.072-4.036) | 0.548 | 0.763 (0.178-3.273) | 0.716 |
| Moderate high | 0.534 (0.356-1.955) | 0.660 | 0.956 (0.424-2.154) | 0.914 |
| High | 0.425 (0.223-0.813) | **0.010** | 0.524 (0.289-0.951) | **0.033** |
| Very high | 0.266 (0.143-0.497) | **<0.001** | 0.429 (0.259-0.712) | **0.001** |

*P* values of <0.05 are statistically significant.

Abbreviations: CI, confidence interval; HR, Hazard ratios; IPSS-M, Molecular International Prognostic Scoring System; LFS, leukemia-free survival; OS, overall survival.

**Supplemental Table 9. Clinical and laboratory characteristics of MDS patients based on IPSS-M in different studies**

| Characteristic | Current study  （n=649） | Junying Wu’s cohort（n=852） | IWG-PM  （n=2,957） | Moffitt Cancer Center  （n=2,355） |
| --- | --- | --- | --- | --- |
| 2022 ICC |  |  |  |  |
| MDS | 485 (74.7) | NA | NA | NA |
| MDS/AML | 164 (25.3) | NA | NA | NA |
| 2016 WHO |  |  |  |  |
| MDS-SLD/MLD | 238 (36.7) | 407 (47.8) | 921 (32) | 788 (33.5) |
| MDS-RS-SLD/MLD | 71 (10.9) | 54 (6.3) | 461 (16) | 347 (14.7) |
| EB1/2 | 329 (50.7) | 358 (42.0) | 887 (31) | 847 (35.9) |
| 5q- syndrome | 4 (0.6) | 12 (1.4) | 142 (4.9) | 97 (4.1) |
| MDS-Unclassified | 7 (1.1) | 21 (2.5) | 85 (3.0) | 48 (2) |
| CMML | 0 (0) | 0 (0) | 272 (9.5) | 0 (0) |
| MDS/MPN-RS-T | 0 (0) | 0 (0) | 42 (1.5) | 0 (0) |
| MDS/MPN-U | 0 (0) | 0 (0) | 51 (1.8) | 0 (0) |
| other | 0 (0) | 0 (0) | 10 (0.3) | 228 (9.7) |
| (Missing) | 0 | 92 | 86 | 0 |
| Type |  |  |  |  |
| Primary | 649 (100%) | 852 (100%) | 2,641 (89.3%) | 1879 (79.8) |
| Secondary | 0 | 0 | 234 (8.1%) | 475 (20.2) |
| (Missing) | 0 | 0 | 82 | 1 |
| Age (years) ^*^ | 67 (53-75) | 56 (44-64) | 72 (63-78) | 70 |
| (Missing) | 0 | 0 | 20 | NA |
| Sex n (%) |  |  |  |  |
| Male | 409 (63) | 550 (64.6) | 1776 (60) | 1484 (63) |
| Female | 240 (37) | 302 (35.4) | 1181 (40) | 871 (37) |
| BM blasts (%) ^*^ | 4.4 (2-10) | 2.5 (1-7) | 3 (1-6) | 4 |
| (Missing) | 0 | 1 | 104 | NA |
| Hemoglobin (g/L) ^*^ | 81 (70-93) | 79 (66-95) | 100 (80110) | 95 |
| (Missing) | 0 | 0 | 105 | NA |
| Platelets (×10^9^/L) ^*^ | 77 (37-150) | 60 (31-119) | 130 (69-236) | 101 |
| (Missing) | 0 | 0 | 118 | NA |
| ANC (×10^9^/L) ^*^ | 1.5 (0.8-3) | 1 (0.7-2) | 2 (1-3) | 1.4 |
| (Missing) | 0 | 0 | 179 | NA |
| IPSS-R karyotype |  |  |  |  |
| Very good | 13 (2.0) | 10 (1.2) | 119 (4.6) | 54 (2.3) |
| Good | 414 (63.8) | 427 (50.1) | 1786 (69) | 1296 (55.0) |
| Intermediate | 98 (15.1) | 186 (21.8) | 356 (14) | 344 (14.6) |
| Poor | 29 (4.5) | 42 (4.9) | 121 (4.7) | 182 (7.7) |
| Very poor | 95 (14.6) | 95 (11.2) | 206 (8.0) | 446 (18.9) |
| (Missing) | 0 | 92 | 369 | 33 |
| IPSS-R |  |  |  |  |
| Very low | 22 (3.4) | 28 (3.3) | 489 (18) | 295 (12.5) |
| Low | 170 (26.2) | 185 (21.7) | 1078 (39) | 671 (28.5) |
| Intermediate | 173 (26.7) | 241 (28.3) | 562 (20) | 442 (18.8) |
| High | 141 (21.7) | 175 (20.5) | 355 (13) | 381 (16.2) |
| Very high | 143 (22.0) | 131 (15.4) | 269 (9.8) | 544 (23.1) |
| (Missing) | 0 | 92 | 204 | 22 |
| Treatment |  |  |  |  |
| HMA | 163 (24.6) | NA | 551 (18.6) | NA |
| Intensive chemo | 21 (3.2) | NA | 60 (2.0) | NA |
| HSCT | 89 (13.4) | NA | 270 (9.1) | NA |
| Reclassification rate | 43 | 41 | 46 | 45 |
| IPSS-M |  |  |  |  |
| Very low | 18 (2.8) | 21 (2.5) | 381 (14.1) | 96 (4.1) |
| Low | 132 (20.3) | 138 (16.2) | 889 (32.9) | 560 (23.8) |
| Moderate low | 95 (14.6) | 125 (14.7) | 302 (11.1) | 327 (13.9) |
| Moderate high | 92 (14.2) | 113 (13.3) | 281 (10.4) | 258 (11.0) |
| High | 121 (18.6) | 170 (20.0) | 379 (14.0) | 454 (19.3) |
| Very high | 191 (29.4) | 192 (22.5) | 469 (17.4) | 660 (28.0) |
| (Missing) | 0 | 93 | 256 | 0 |
| Median LFS (years) | *P*<0.001 | *P*<0.001 | *P*<0.001 | NA |
| Very low | 13.0 | NA | 9.7 | 12.3 |
| Low | 15.5 | NA | 6. | 6.9 |
| Moderate low | 7.1 | NA | 4. | 3.6 |
| Moderate high | 4.2 | NA | 2.3 | 2.2 |
| High | 2.1 | NA | 1.5 | 1.4 |
| Very high | 0.7 | NA | 0 | 0.5 |
| Median OS (years) | *P*<0.001 | *P*<0.001 | *P*<0.001 | NA |
| Very low | 13.0 | NA | 10.6 | 11.7 |
| Low | 15.5 | NA | 6.0 | 7.1 |
| Moderate low | 7.1 | NA | 4.6 | 4.4 |
| Moderate high | 4.8 | NA | 2.8 | 3.1 |
| High | 2.6 | NA | 1.7 | 2.3 |
| Very high | 1.0 | NA | 1.0 | 1.3 |

*Median (inter-quartile ranges).

Abbreviations: ANC, absolute neutrophil count; BM, bone marrow; CMML, chronic myelomonocytic leukemia; chemo, chemotherapy; EB, excess blasts; HMA, hypomethylating agents; HSCT, hematopoietic stem cell transplantation; IWG-PM, International Working Group for Prognosis in MDS; ICC, International Consensus Classification; IPSS-M, Molecular International Prognostic Scoring System; IPSS-R, Revised International Prognostic Scoring System; LFS, leukemia-free survival; MDS, myelodysplastic syndromes; MDS/AML, myelodysplastic syndromes/acute myeloid leukemia; MPN, myeloproliferative neoplasm; MLD, multilineage dysplasia; MDS/MPN-RS-T, MDS/MPN with ring sideroblasts and thrombocytosis; MDS/MPN-U, MDS/MPN-unclassifiable; NA, not applicable; OS, overall survival; RS, ring sideroblasts; SLD, single-lineage dysplasia; WHO, World Health Organization

**Supplemental Figure 1. Mutation frequencies for 649 patients with myelodysplastic syndromes.**

Note: *ETNK1, GNB1, NF1, PPM1D*, and *PRPF8* were not included.

**Supplemental Figure 2. Distribution of the IPSS-M, IPSS-R and IPSS based on myelodysplastic syndromes or myelodysplastic syndromes/acute myeloid leukemia**

(a-c) Distribution of the IPSS-M (a)/IPSS-R (b)/IPSS (c) in patients with MDS or MDS/AML

(d-f) Distribution of the IPSS-M (d)/IPSS-R (e)/IPSS (f) in patients with different subtypes of MDS per International Consensus Classification

(g-i) Distribution of the IPSS-M (g)/IPSS-R (h)/IPSS (i) in patients with different subtypes of MDS/AML per International Consensus Classification

| (a) | (b) | (c) |
| --- | --- | --- |
|  |  |  |
| (d) | (e) | (f) |
| *SF3B1*  *TP53* | *SF3B1*  *TP53* | *SF3B1*  *TP53* |
|  |  |  |
|  |  |  |
|  |  |  |
| (g) | (h) | (i) |
| *TP53* | *TP53* | *TP53* |

Abbreviations: EB, excess blasts; IPSS, International Prognostic Scoring System; IPSS-M, Molecular International Prognostic Scoring System; IPSS-R, Revised International Prognostic Scoring System; MDS, myelodysplastic syndromes; MDS/AML, myelodysplastic syndromes/acute myeloid leukemia; MLD, multilineage dysplasia; NOS, not otherwise specified; SLD, single lineage dysplasia.

**Supplemental Figure 3. Mutation frequencies for patients who may receive disease-modifying treatments (n=110) after reclassification by the IPSS-M.**

|  |
| --- |

Note: *ETNK1, GNB1, NF1, PPM1D*, and *PRPF8* were not included.

**Supplemental Figure 4. Kaplan-Meier curves for leukemia-free survival and overall survival in patients with myelodysplastic syndromes or myelodysplastic syndromes/acute myeloid leukemia, categorized by the IPSS-M**

(a,b) Leukemia-free survival (a) and overall survival (b) for patients with MDS, categorized by the IPSS-M

(c,d) Leukemia-free survival (c) and overall survival (d) for patients with MDS/AML, categorized by the IPSS-M

| (a) | (b) |
| --- | --- |
| 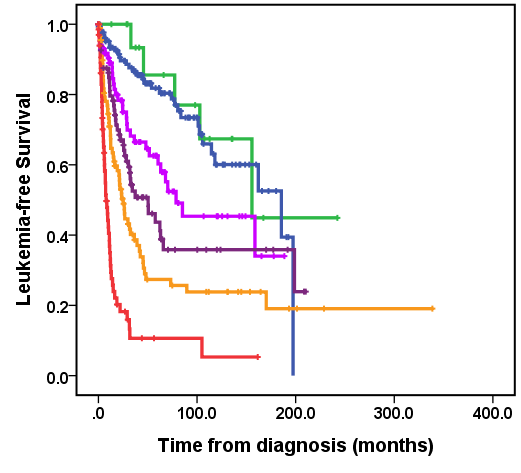  **Moderate high-risk IPSS-M, n=84**  **Low-risk IPSS-M, n=132**  **High-risk IPSS-M, n=92**  **Very low-risk IPSS-M, n=18**  **Very high-risk IPSS-M, n=68**  **Moderate low-risk IPSS-M, n=91**  ***P* = 0.546**  ***P* = 0.003**  ***P* = 0.114**  ***P* = 0.046**  ***P* < 0.001** | 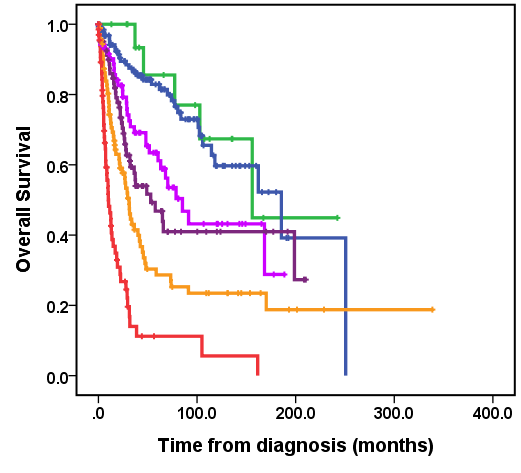  **Moderate high-risk IPSS-M, n=84**  **Low-risk IPSS-M, n=132**  **High-risk IPSS-M, n=92**  **Very low-risk IPSS-M, n=18**  **Very high-risk IPSS-M, n=68**  **Moderate low-risk IPSS-M, n=91**  ***P* = 0.680**  ***P* = 0.004**  ***P* = 0.288**  ***P* = 0.020**  ***P* < 0.001** |
| (c) | (d) |
| 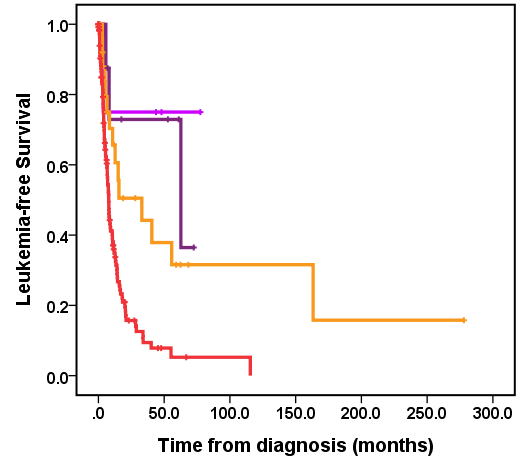  **Moderate high-risk IPSS-M, n=8**  **High-risk IPSS-M, n=29**  **Very high-risk IPSS-M, n=123**  **Moderate low-risk IPSS-M, n=4**  ***P* = 0.086**  ***P* = 0.288**  ***P* = 0.001** | 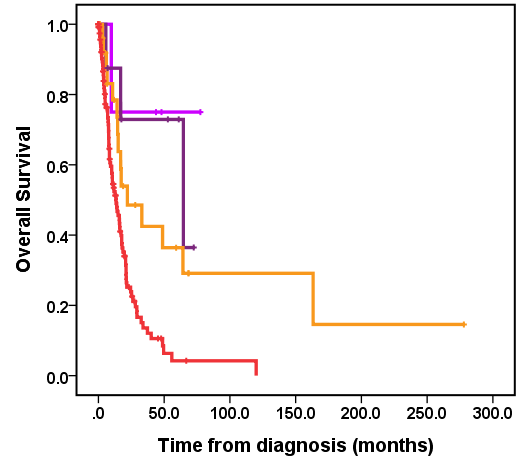  **Moderate high-risk IPSS-M, n=8**  **High-risk IPSS-M, n=29**  **Very high-risk IPSS-M, n=123**  **Moderate low-risk IPSS-M, n=4**  ***P* = 0.674**  ***P* = 0.301**  ***P* = 0.002** |

Abbreviations: IPSS-M, Molecular International Prognostic Scoring System; MDS, myelodysplastic syndromes; MDS/AML, myelodysplastic syndromes/acute myeloid leukemia.

**Supplemental Figure 5. Kaplan-Meier curves of leukemia-free survival and overall survival for patients with myelodysplastic syndromes, reclassified by the IPSS-M (down-stage *vs.* unchanged *vs*. up-stage) within each IPSS-R group**

(a,b) Leukemia-free survival (a) and overall survival (b) for patients with very low/low-risk IPSS-R

(c,d) Leukemia-free survival (c) and overall survival (d) for patients with intermediate-risk IPSS-R

(e,f) Leukemia-free survival (e) and overall survival (f) for patients with high-risk IPSS-R

(g,h) Leukemia-free survival (g) and overall survival (h) for patients with very high-risk IPSS-R

| (a) | (b) |
| --- | --- |
| 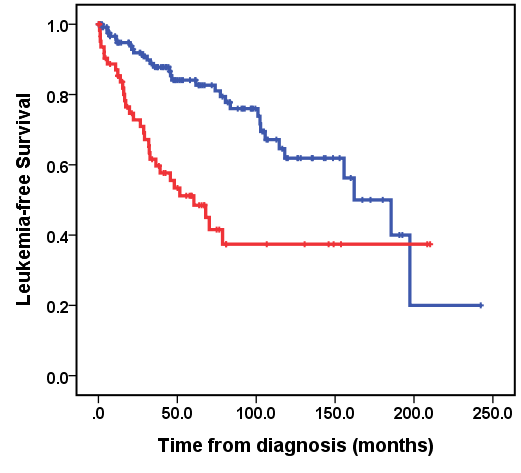  **Unchanged, n=127**  **Up-stage, n=65**  ***P* < 0.001** | 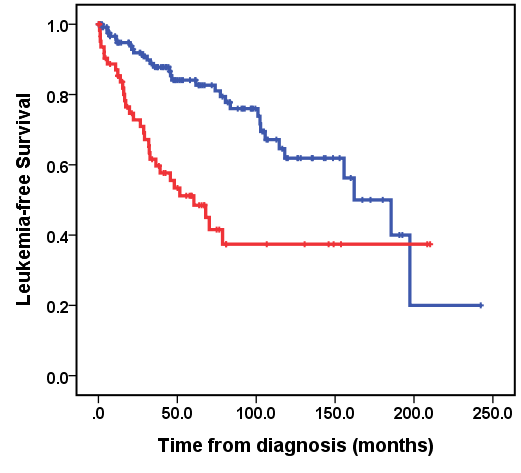  **Unchanged, n=127**  **Up-stage, n=65**  ***P* < 0.001** |
| (c)  **Down-stage, n=23**  **Unchanged, n=102**  **Up-stage, n=48**  ***P* = 0.161**  ***P* < 0.001** | (d)  **Down-stage, n=23**  **Unchanged, n=102**  **Up-stage, n=48**  ***P* = 0.241**  ***P* < 0.001** |
| 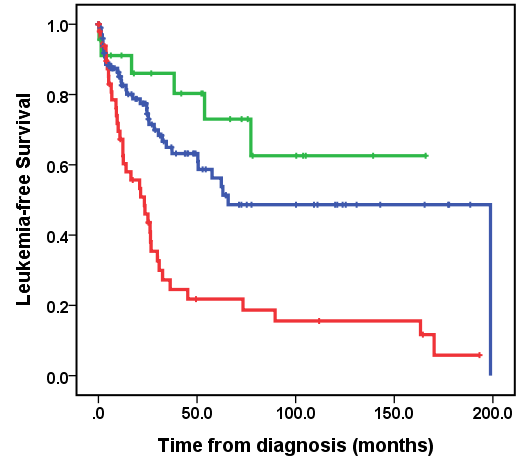 | 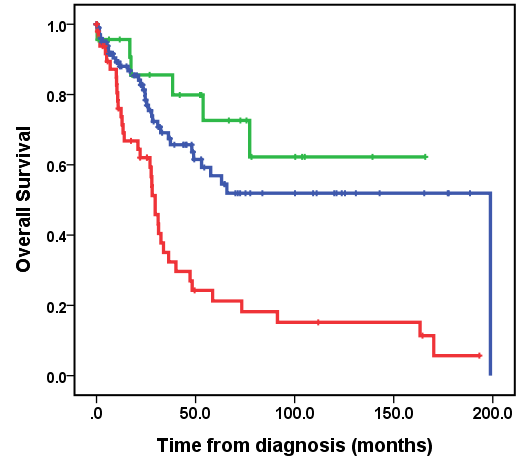 |
| (e) | (f)  **Down-stage, n=29**  **Unchanged, n=48**  **Up-stage, n=64**  ***P* = 0.075**  ***P* < 0.001** |
| 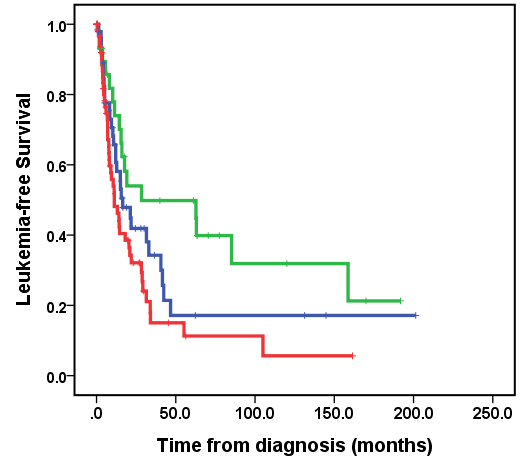  **Down-stage, n=29**  **Unchanged, n=48**  **Up-stage, n=64**  ***P* = 0.186**  ***P* = 0.089** | 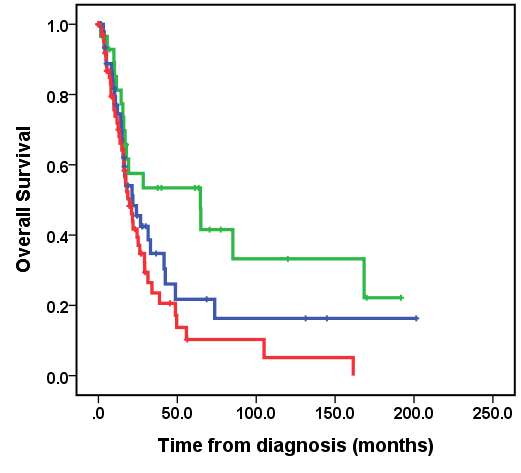 |

| (g) | (h) |
| --- | --- |
| 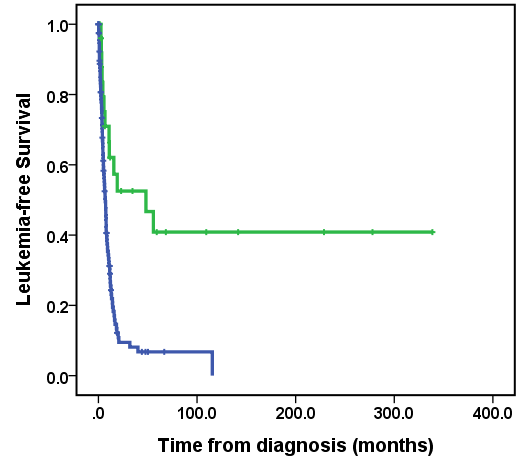  ***P* < 0.001**  **Unchanged, n=118**  **Down-stage, n=25** | 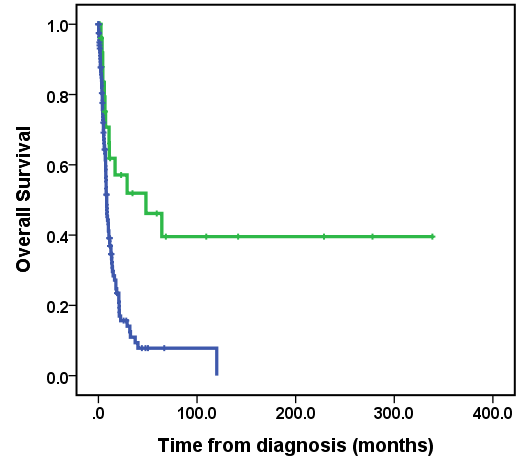  ***P* < 0.001**  **Unchanged, n=118**  **Down-stage, n=25** |

Abbreviations: IPSS-M, Molecular International Prognostic Scoring System; IPSS-R, Revised International Prognostic Scoring System.

**Supplemental Figure 6. Kaplan-Meier curves of leukemia-free survival and overall survival for patients with myelodysplastic syndromes, classified by the IPSS-R within each IPSS-M group**

(a,b) Leukemia-free survival (a) and overall survival (b) for patients with very low/low-risk IPSS-M

(c,d) Leukemia-free survival (c) and overall survival (d) for patients with moderate low-risk IPSS-M

(e,f) Leukemia-free survival (e) and overall survival (f) for patients with moderate high-risk IPSS-M

(g,h) Leukemia-free survival (g) and overall survival (h) for patients with high-risk IPSS-M

(i,j) Leukemia-free survival (i) and overall survival (j) for patients with very high-risk IPSS-M

| (a) | (b) |
| --- | --- |
| 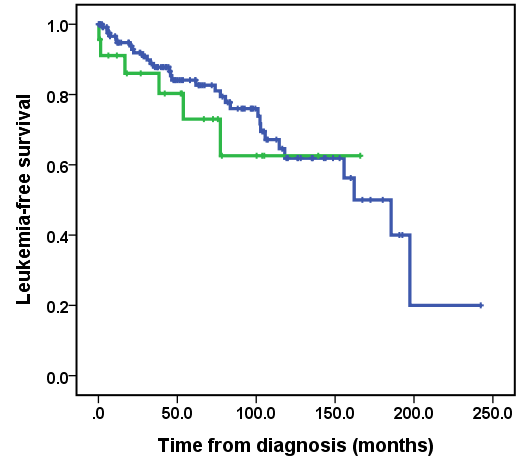  **Very low/low risk IPSS-R, n=127**  **Intermediate risk IPSS-R, n=23**  ***P* = 0.467** | 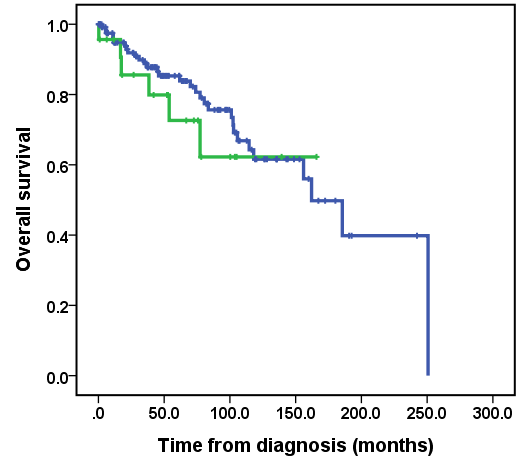  **Very low/low risk IPSS-R, n=127**  **Intermediate risk IPSS-R, n=23**  ***P* = 0.480** |
| (c)  **Up-stage, n=6**  **Unchanged, n=48**  **Down-stage, n=41**  ***P* = 0.535**  ***P* = 0.267** | (d) |
| 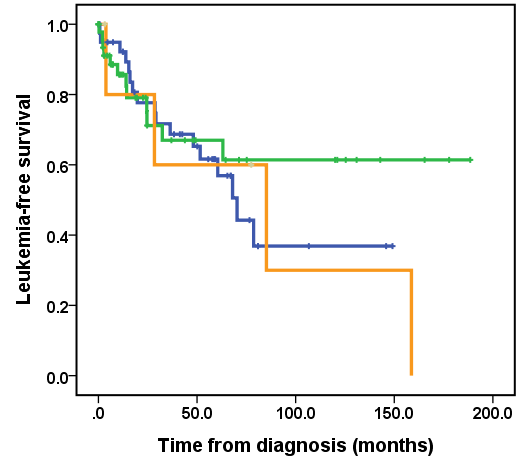  **Intermediate risk IPSS-R, n=48**  ***P* = 0.557**  **High risk IPSS-R, n=6**  **Very low/low risk IPSS-R, n=41** | 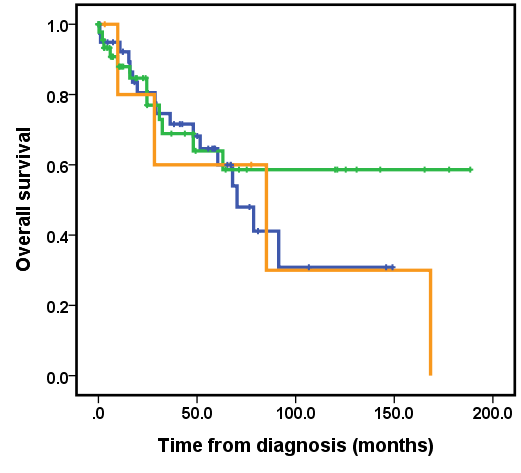  **Intermediate risk IPSS-R, n=48**  **Very low/low risk IPSS-R, n=41**  ***P* = 0.571**  **High risk IPSS-R, n=6** |
| (e) | (f) |
| 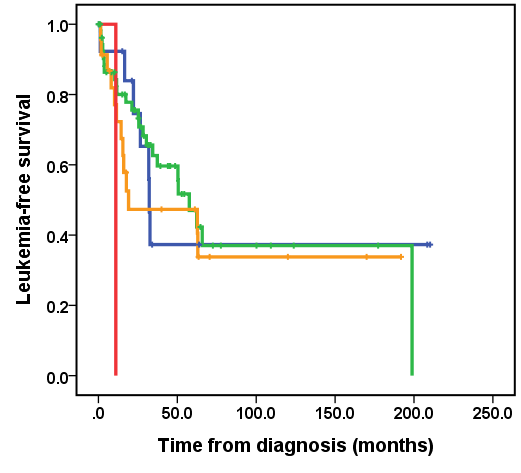  **Very low/low risk IPSS-R, n=14**  **Intermediate risk IPSS-R, n=54**  **High risk IPSS-R, n=23**  ***P* = 0.237**  **Very high risk IPSS-R, n=1** | 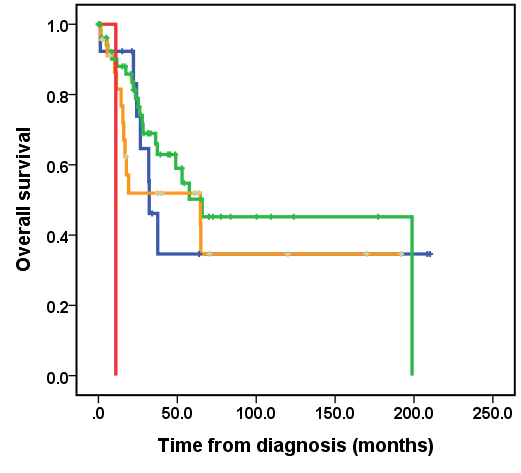  **Very low/low risk IPSS-R, n=14**  **Intermediate risk IPSS-R, n=54**  ***P* = 0.057**  **Very high risk IPSS-R, n=1**  **High risk IPSS-R, n=23** |
|  |  |
|  |  |
|  |  |
|  |  |
|  |  |
| (g) | (h) |
| 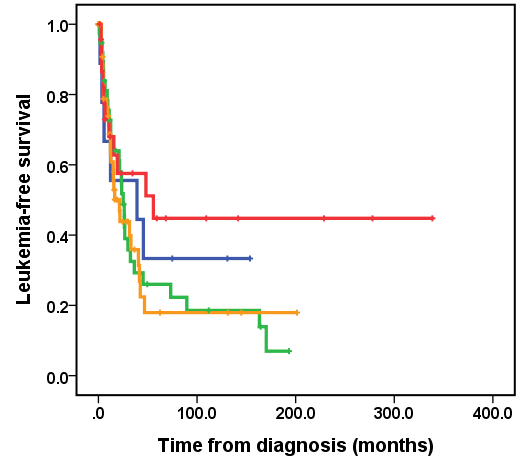  **Very low/low risk IPSS-R, n=9**  **Very high risk IPSS-R, n=24**  ***P* = 0.338**  **High risk IPSS-R, n=48**  **Intermediate risk IPSS-R, n=40** | 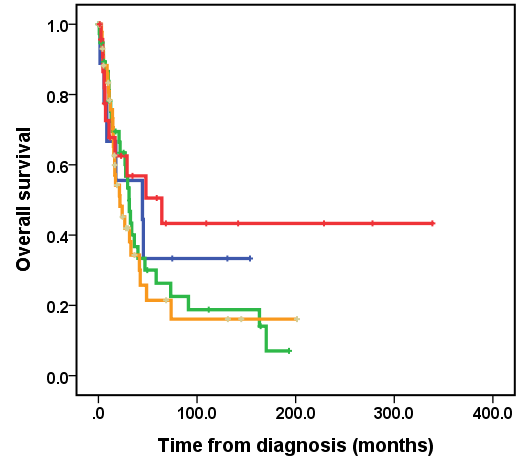  **Very low/low risk IPSS-R, n=9**  **Very high risk IPSS-R, n=24**  **Intermediate risk IPSS-R, n=40**  **High risk IPSS-R, n=48**  ***P* = 0.376** |
| (i) | (j) |
| 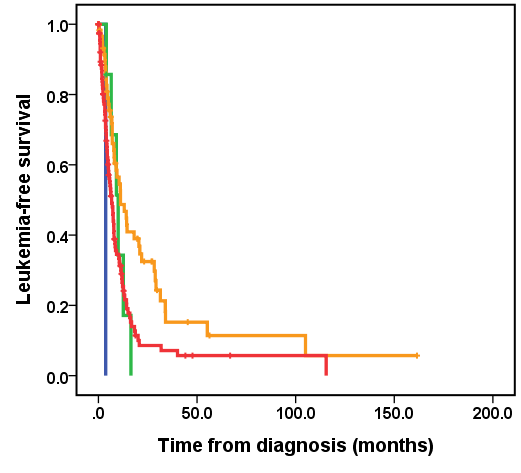  **Very low/low risk IPSS-R, n=1**  **Intermediate risk IPSS-R, n=8**  ***P* = 0.003**  **Very high risk IPSS-R, n=118**  **High risk IPSS-R, n=64** | 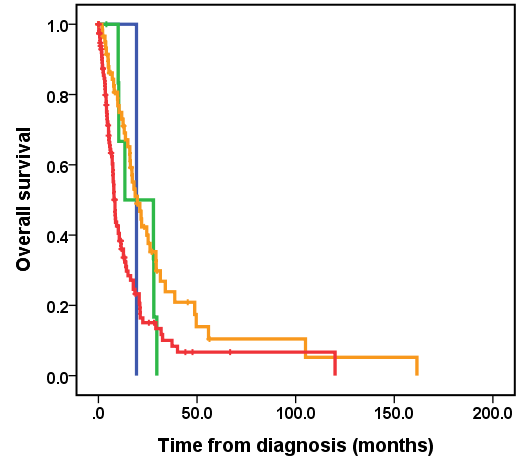  **Very low/low risk IPSS-R, n=1**  **Intermediate risk IPSS-R, n=8**  ***P* = 0.002**  **Very high risk IPSS-R, n=118**  **High risk IPSS-R, n=64** |

Abbreviations: IPSS-M, Molecular International Prognostic Scoring System; IPSS-R, Revised International Prognostic Scoring System.

**Supplemental Figure 7. Impacts of hematopoietic stem cell transplantation on leukemia-free survival and overall survival in patients with high or very high-risk IPSS-M**

(a,b) Leukemia-free survival (a) and overall survival (b) of patients receiving or not receiving hematopoietic stem cell transplant in those with high-risk IPSS-M

(c,d) Leukemia-free survival (c) and overall survival (d) of patients receiving or not receiving hematopoietic stem cell transplant in those with very high-risk IPSS-M

| (a) | (b) |
| --- | --- |
| 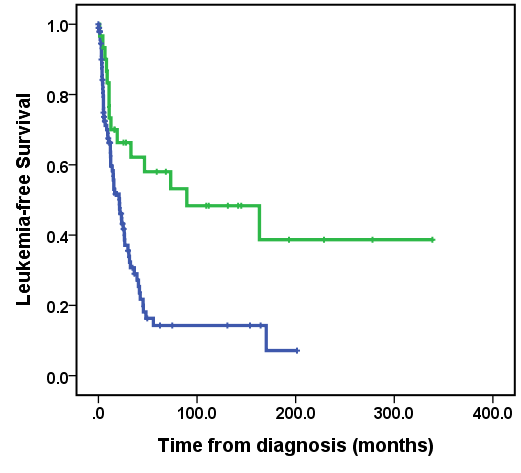  ***P* = 0.001**  **HSCT (-), n=95**  **HSCT (+), n=27** | 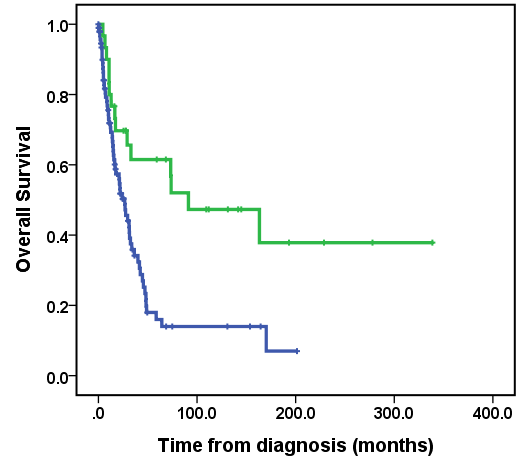  ***P* = 0.001**  **HSCT (-), n=95**  **HSCT (+), n=27** |
| (c) | (d) |
| 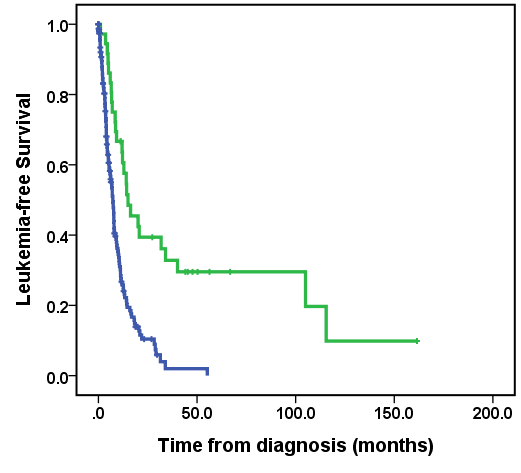  ***P* < 0.001**  **HSCT (-), n=156**  **HSCT (+), n=35** | 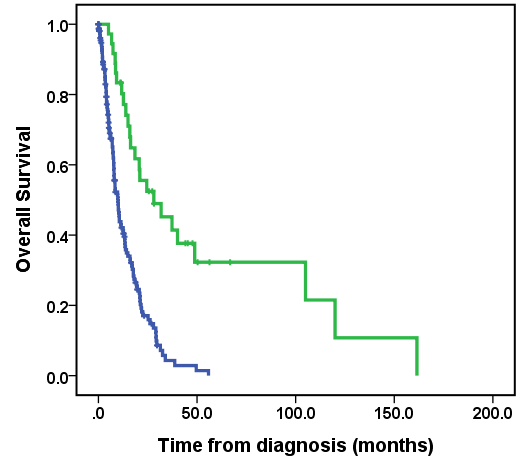  ***P* < 0.001**  **HSCT (-), n=156**  **HSCT (+), n=35** |

Abbreviations: HSCT, allogeneic hematopoietic stem cell transplantation; IPSS-M, Molecular International Prognostic Scoring System; IPSS-R, Revised International Prognostic Scoring System

**Supplemental Figure 8. Kaplan-Meier curves for leukemia-free survival and overall survival, stratified based on *SF3B1* mutation status**

(a) Leukemia-free survival, stratified by *SF3B1* mutation status

(b) Overall survival, stratified by *SF3B1* mutation status

| (a)  **Wild type *SF3B1*, n=558**  ***SF3B1*^α^, n=70**  ***SF3B1*^β^, n=18**  ***SF3B1*^5q^, n=2**  ***P* = 0.016**  ***P* = 0.004**  ***P* = 0.042** | (b)  **Wild type *SF3B1*, n=558**  ***SF3B1*^α^, n=70**  ***SF3B1*^β^, n=18**  ***SF3B1*^5q^, n=2**  ***P* = 0.038**  ***P* = 0.012**  ***P* = 0.042** |
| --- | --- |
| 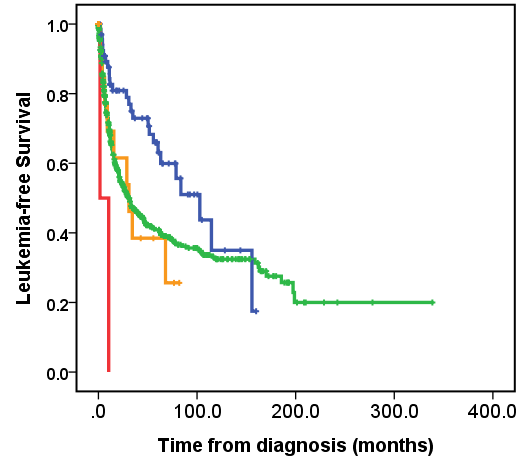 | 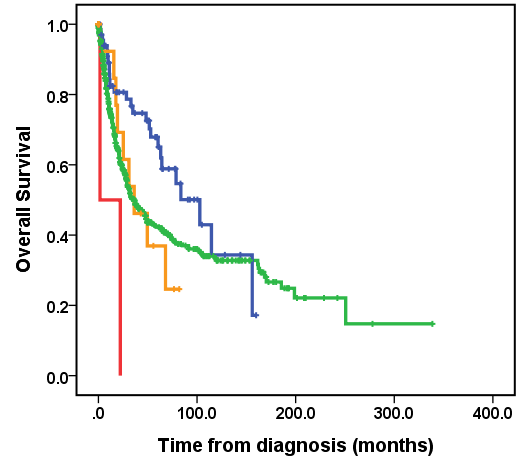 |

Note: *SF3B1*^5q^: concomitant presence with isolated del(5q); *SF3B1*^β^: comutation between *SF3B1* and any gene from *BCOR, BCORL1, NRAS, RUNX1, SRSF2*, or *STAG2*; *SF3B1*^α^: as any other mutant *SF3B1*
